# Thiol/Disulfide Homeostasis in Pericardial Fluid and Plasma of Patients Undergoing Coronary Artery Bypass Surgery

**DOI:** 10.21470/1678-9741-2022-0367

**Published:** 2025-02-14

**Authors:** Reşat Dikme, Abdullah Taşkin

**Affiliations:** 1 Department of Medical Biology and Genetics, Vocational School of Health Services, Harran University, Şanliurfa, Turkey; 2 Department of Nutrition and Dietetics, Faculty of Health Sciences, Harran University, Şanliurfa, Turkey.

**Keywords:** Coronary Artery Bypass, Disulfides, Extracorporeal Circulation, Pericardia Fluid, Homeostasis, Indicators and Reagents, Ischemia, Oxidation-Reduction, Reperfusion, Sulfhydryl Compounds

## Abstract

**Introduction:**

On-pump coronary artery bypass grafting (CABG) method affect almost allbiochemicalreactions by disrupting the patient’s redox homeostasis. Detection of systemic redox hemostasis in the patient is critical for the CABG method’s success and the prognosis of the disease. In this study, thiol/disulfide parameters, which are indicators of redox homeostasis, and ischemia-modified albumin levels in the plasma and pericardial fluid of patients who underwent CABG were investigated.

**Methods:**

Sixty patients who underwent an on-pump CABG operation with the cardiopulmonary bypass method were included in this study. Blood samples were taken from the patients before and after cardiopulmonary bypass. Pericardia fluid samples were taken before cardiopulmonary bypass. Then, thiol/disulfide homeostasis, albumin, and ischemia-modified albumin levels in the pericardial fluid and the patients’ plasma levels were compared.

**Results:**

Albumin and ischemia-modified albumin levels were significantly higher in the postoperative period compared to the preoperative one (P<0.001). Thiol/disulfide parameters were higher and statistically significant in preoperative than in postoperative examinations (*P*<0.001). A negative correlation was found between pericardial fluid ischemia-modified albumin and thiol-disulfide parameters (*P*<0.001).

**Conclusion:**

Changes in thiol/disulfide homeostasis, albumin, and ischemia-modified albumin levels at different times during the on-pump CABG may be caused by foreign non-endothelial surfaces, filters, the reperfusion process, and pharmacological effects in the extracorporeal circulation. Thiol/disulfide homeostasis, albumin, and ischemia-modified albumin levels should be monitored during the on-pump CABG and should be intervened with appropriate therapeutic strategies. In this way, secondary pathologies can be avoided by preventing cellular damage and excessive inflammatory responses.

## INTRODUCTION

Reactive oxygen species (ROS) and oxidative stress have negative effects on processes such as the development and acceleration of coronary artery disease (CAD) and plaque formation^[1,2]^. In particular,

ROS, which is formed by the deterioration of molecular and cellular functions, causes oxidative damage above physiological levels^[3]^. High oxidative stress, which occurs as a result of decreased antioxidants and increased oxidants together with inflammation, has a synergistic effect on the standard risk factors of CAD^[4]^. Therefore, the delicate balance between oxidants and antioxidants is vital in these processes.

The main goal in ROS-mediated biochemical reactions is not to create oxidative stress, but to try to maintain and restore “redox homeostasis”^[5]^. Thiol/disulfide level has critical importance in maintaining plasma and intracellular redox homeostasis^[5,6]^. Thiols, which are the main factor in ensuring the redox balance, have a high sensitivity to oxidation due to the -SH (one sulfur and one hydrogen atom) groups in their structures and interact with almost all physiological oxidants. Because of these properties, they are considered “essential antioxidant buffers”^[6,7,8,9]^. In addition to their antioxidant properties, they play a role in many biochemical events such as regulation of protein functions, signal transduction, regulation of transcription factors, and immune response^[9,10,11]^. Disulfides, the oxidized form of thiols, are redox-sensitive covalent bonds formed between two thiol groups (sulfhydryl atom). Disulfide bond structures formed by ROS oxidation can revert to thiol groups and thus maintain the thiol/disulfide balance. The thiol/disulfide ratio is a new marker used as a measure of thiol and disulfide homeostasis. Thiol/disulfide levels change significantly in the pathogenesis of cardiovascular diseases, diabetes, cancer, and renal failure^[6]^.

The N-terminal amino end of the albumin molecule, which is an important source of thiol and accepted as the thiol pool of the plasma, is the binding site of metal ions such as Co^+2^, Ni^+2^, and Cu+2. In oxidative stress, which occurs in many different conditions such as acidosis, hypoxia, and exposure to free iron and copper, the N-terminus of albumin is modified and its ion-binding ability decreases^[12]^. This modified form of albumin is called ischemia-modified albumin (IMA)^[13]^. In various coronary syndromes, ischemia, acidosis, hypoxia, and ROS increase caused by decreased coronary blood flow lead to albumin modification and an increased IMA level^[14]^. IMA levels, which increase in a short time in the early phase of ischemia and in myocardial infarction, are used as a cardiac biomarker in clinical practice^[15,16]^. IMA, which reflects myocardial ischemia within minutes, shows the degree of short-term oxidative effect^[17]^. Sinha et al.^[18]^ reported that the sensitivity of the IMA level increased to 95% when used together with electrocardiography and cardiac troponin (or cTnT) in acute coronary syndrome.

Foreign material surface, hemolysis, surgical procedure, and reperfusion affect the cellular redox balance in the on-pump coronary artery bypass grafting (CABG) method, which is used for surgical treatment in CAD and performed with the help of cardiopulmonary bypass (CPB). In addition, the high level of molecular oxygen given to the circulatory system during on-pump CABG creates cellular stress and activates the inflammatory system. The resulting stress and inflammatory response affect almost all biochemical reactions by disrupting holistic homeostasis and causing serious damage^[19,20]^. A shift in the thiol/disulfide redox balance at this stage can have adverse systemic consequences. Therefore, detection of thiol/disulfide homeostasis is extremely important in terms of controlling redox-mediated inflammatory signaling pathways during on-pump CABG. Although there are many studies on thiol/disulfide homeostasis, there are few studies on the relationship between on-pump CABG and thiol/disulfide homeostasis and IMA levels. In addition, such a study related to the subject of pericardial fluid (PF) has not been found in the literature. Thanks to this study, it is important to investigate the thiol/disulfide balance and IMA levels in both the PF and plasma of the same patient (before and after on-pump CABG) in terms of their secondary effects. In this study, the physiological and biochemical changes of the heart in terms of thiol/disulfide balance were investigated by examining the PF, which is the closest tissue fluid to the heart, and gives accurate information about the heart.

Plasma thiol/disulfide ratio can be an easy target for therapeutic intervention by N-acetylcysteine or other thiol compounds. In order to control the oxidative stress that may occur during on-pump CABG, thiol/disulfide homeostasis can be followed to prevent negative situations that may be caused by stress. For this, many treatment strategies can be developed, including the addition of antioxidant substances to the CPB system or intrapericardial drug administration.

In this study, preoperative and postoperative thiol/disulfide balance and IMA levels were compared in the plasma of patients undergoing CABG. In addition, the thiol/disulfide balance and IMA levels in PF were also investigated in this study and their relationship with plasma was evaluated.

## METHODS

### Study Population and Processes

A total of 60 patients who underwent on-pump CABG were included in this study. The present study was approved by the local ethics committee (approval number: 04.07.2022-2022/13/19). All operations were carried out by the same operations team. Exclusion criteria consisted of patients who had a preoperative infection, had a blood product transfusion, received inotropic support, had a history of oxygen support in the last two weeks, and needed postoperative extracorporeal membrane oxygenation support. All procedures were performed under general anesthesia with mild hypothermic CPB. Intermittent isothermal blood cardioplegia was used for myocardial protection. General anesthesia was maintained with sevoflurane and intravenous rocuronium was used for neuromuscular blockade.

### Cardiopulmonary Bypass Management

Standard CPB was performed with mild hypothermia (32°C). After median sternotomy and heparinization (300 IU/kg), CPB was performed with aorto-venous two-stage cannulation. A cross-clamp was placed on the ascending aorta, and cardiac arrest was provided with antegrade cardioplegia (10 mL/kg) with high potassium. Continuity of the cardiac arrest was provided with blood cardioplegia given every 20-30 minutes. CPB was established with a roller pump with a membrane oxygenator (Maquet, Getinge Group, Restalt, Germany) and arterial line filter at pump flow rates of 2.2-2.4 L/min/m^2^.

### Pericardial Fluid and Blood Sample Collection

Before and after on the CPB, we collected venous blood samples from all patients. The collected samples were centrifuged at 1500 g for 10 minutes at 4°C and stored at −80°C until analysis. In addition, after median sternotomy was performed with standard CPB procedures, the pericardium was opened, and the PF was aspirated with a sterile syringe. The aspirated PF (2-5 mL) was then taken into sterile tubes without anticoagulant and placed in an ice-filled container. Afterward, the samples were centrifuged (2500 g for five minutes at 4°C) two times, and the supernatant phase was separated. Supernatants were stored in RNase-free tubes at -80°C until assayed. Albumin levels in plasma and PF samples were measured by the biuret method.

### Measurement of Thiol/Disulfide Homeostasis

The thiol/disulfide homeostasis parameters were studied with a new method previously described by Erel et al.^[6]^ First, dynamic disulfide bonds (-S-S) were reduced to reactive thiol groups in the presence of sodium borohydride. Later, total thiol and native thiol (-SH) levels were determined using Ellman reagents^[21]^. The dynamic disulfide amount was obtained by dividing the difference between the total and native thiol by two. Oxidized thiol, reduced thiol, and thiol-oxidation reduction parameters were calculated according to the formula:

Oxidized thiol [(-S-S-) X 100/total thiol)], reduced thiol [(-SH X 100)/total thiol], and thiol oxidation reduction [(-S-S-) X 100/(-SH)]

Native thiol, total thiol, and disulfide levels were expressed as µmol/L.

### Measurement of Ischemia-Modified Albumin

Plasma and PF IMA levels were analyzed using the colorimetric method developed by Bar-Or et al.^[22]^. In the basic principle of the test, 50 µL of cobalt chloride reagent is added to 200 µL of plasma/PF. The mixture is incubated for 10 minutes to form the albumincobalt complex. Then, 50 µL (1.5 mg/mL) of dithiothreitol solution is added as a coloring agent and vortexed. Finally, 1.0 mL of 0.9% NaCl is added. The colored complex formed by the addition of NaCl is measured spectrophotometrically at a wavelength of 470 nm. Results are expressed in absorbance units.

### Statistical Analysis

In assessing the data, IBM Corp. Released 2017, IBM SPSS Statistics for Windows, version 25.0, Armonk, NY: IBM Corp. statistical package program has been used. Shapiro-Wilk test was used to control the normal distribution of data. Data with and without normal distribution were expressed as mean ± standard deviation and median (range of quarters), respectively. Paired-samples f-test (in parametric assumptions) and Wilcoxon test (for non-parametric assumptions) were used to investigate the relationship between the two dependent variables. The relationship between the parameters was made using Pearson’s or Spearman’s correlation analysis. *P*<0.05 was regarded as statistically significant.

## RESULTS

The demographic characteristics of the patients are summarized in Table 1. Twenty-four of the patients were female and 36 were male, with a mean age of 61.38 ± 10.55 years, the height of 164.17 ± 6.82 cm, the weight of 77.42 ± 18.32 kg, and body surface area of 1.84 ± 0.23 m^2^.

**Table 1:** Demographic characteristics of patients (n=60).

Demographic findings	n	%	Mean ± SD
Age (years)	60		61.38 ± 10.55
Sex (male/female)	36/24	60/40	
Body surface area (m^2^)			1.84 ± 0.23
Number of anastomoses	-		2.63 ± 0.80
Height (cm)			164.17 ± 6.2
Weight (kg)			77.42 ± 18.2

SD=standard deviation

Number of anastomoses: number of connected vessels from the aorta to occluded coronary arteries

**Fig. 1:**
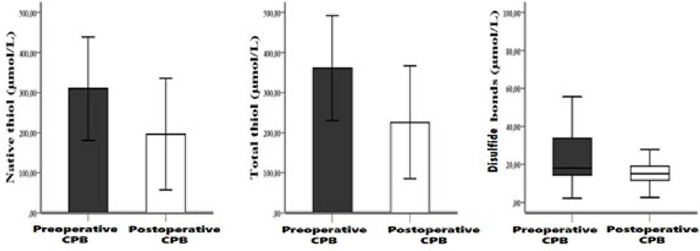
Plasma native thiol, total thiol, and disulfide bonds levels In preoperative and postoperative cardiopulmonary bypass (CPB) (P<J.001, P<0.001, and P=0.001, respectively).

Table 2 shows the albumin, IMA, and thiol/disulfide homeostasis levels of plasma samples taken before and after CPB. Our results showed that albumin and IMA levels were significantly higher postoperatively compared to preoperatively: 5.32 (0.62), 0.80 ± 0.09, and 4.19 (1.55), 0.71 ± 0.10, respectively (*P*<0.001, *P*<0.001). In comparison, thiol/disulfide parameters, native thiol, total thiol, and disulfide levels were higher and more statistically significant preoperatively than postoperatively (*P*<0.001, *P*<0.001, *P*=0.001, respectively) (Figure 1).

**Table 2:** Plasma thiol/disulfide homeostasis in patients undergoing CPB surgerya.

Variables	Preoperative CPB (n = 60)	Postoperative CPB (n = 60)	P-value^b^
Albumin (gr/dL)	4.19 (1.55)	5.32 (0.62)	< 0.001^d^
IMA (ABSU)	0.71 ± 0.10	0.80 ± 0.09	< 0.001^c^
Native thiol (µmol/L)	310.28 ± 64.78	197.38 ± 69.72	< 0.001^c^
Total thiol (µmol/L)	365.20 (66.28)	233.18 (81.16)	< 0.001^d^
Disulfide bonds (µmol/L)	17.99 (20.01)	15.15 (8.51)	0.001^d^
Reduced thiol (%)	90.00 (13.63)	86.52 (8.11)	0.686^d^
Oxidized thiol (%)	4.99 (6.81)	6.90 (3.89)	0.653^d^
Thiol oxidation-reduction (%)	5.55 (9.43)	7.82 (5.26)	0.696^d^

^a^Data are expressed as mean ± standard deviation and median (interquartile range) where appropriate;^b^ *P*<0.05 was considered significant; Obtained from paired samples *t*-test; ^d^Obtained from Wilcoxon test

ABSU=absorbance units; CPB=cardiopulmonary bypass; IMA=ischemia-modified albumin

**Fig. 2:**
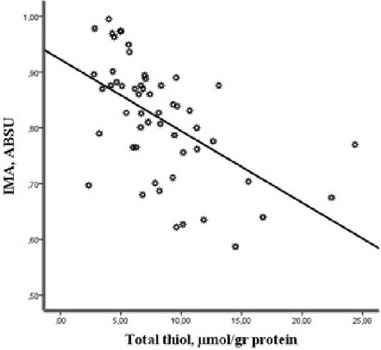
Correlative relationship between pericardial fluid ischemia-modified albumin (IMA) and total thiol levels (r= -0.552, P< 0.001). ABSU=absorbance units.

There was no statistical difference between the preoperative and postoperative periods in terms of reduced thiol, oxidized thiol, and thiol oxidation-reduction (*P*>0.05 for all). Table 3 shows the thiol/disulfide parameters, IMA, and albumin levels in the PF. There was a negative correlation between PF IMA and thiol/disulfide parameters (IMA *vs.* native thiol, *r* = −0.530, *P*<0.001; IMA *vs.* total thiol, *r* = −0.552, *P*<0.001; IMA *vs.* disulfide, *r* = −0.517, *P*<0.001) (Figure 2).

**Table 3:** Pericardial fluid thiol/disulfide homeostasis in patients undergoing CPB surgery^a^.

Variables	CPB (n = 60)
Albumin (gr/dL)	2.55 ± 0.73
IMA (ABSU)	0.81 ± 0.10
Native thiol (µmol/gr protein)	5.41 (3.76)
Total thiol (µmol/gr protein)	7.01 (4.72)
Disulfide bonds (µmol/gr protein)	0.78 (0.67)
Reduced thiol (%)	74.80 ± 7.44
Oxidized thiol (%)	12.59 ± 3.72
Thiol oxidation-reduction (%)	16.36 (11.66)

^a^Data are expressed as mean ± standard deviation and median (interquartile range) where appropriate ABSU=absorbance units; CPB=cardiopulmonary bypass; IMA=ischemia-modified albumin

The results of the Pearson’s and Spearman’s correlation analysis investigating the association between PF IMA, native thiol, total thiol, and disulfide bonds and serum preoperative-postoperative parameters are shown in Table 4. There was no correlation between preoperative and postoperative PF levels.

**Table 4:** Pearson’s and Spearman’s correlation analysis between pericardial fluid parameters and serum preoperative-postoperative parameters.

	Pericardial fluid	Preoperative CPB (n = 60)	Postoperative CPB (n = 60)
IMA	*r*	−0.040^a^	−0.047^a^
	*P*	0.775	0.737
Native thiol	*r*	−0.132^a^	−0.040^a^
	*P*	0.346	0.776
Total thiol	*r*	−0.082^b^	0.081^b^
	*P*	0.557	0.559
Disulfide bonds	*r*	−0.078^b^	0.173^b^
	*P*	0.580	0.211

^a^Obtained from Pearson’s correlation analysis; ^b^Obtained from Spearman’s correlation analysis CPB=cardiopulmonary bypass; IMA=ischemia-modified albumin

## DISCUSSION

During the CPB performed with an on-pump CABG, tissue perfusion and blood pressure deteriorate, blood flow decreases, and ischemia, acidosis, hypoxia, and ROS increase with the effect of foreign material surface^[23]^. Oxidative stress caused by all these multifactorial factors increases myocardial damage, triggers inflammatory processes, limits the success of the surgical procedure, and adversely affects the patient’s survival or recovery time. Therefore, detection of systemic redox hemostasis before and after on-pump CABG is extremely important for the prognosis of the surgical procedure and the disease. The results obtained in the study show that the levels of native thiol, total thiol, and disulfide decrease after an on-pump CABG.

The non-specific inflammatory response in the CPB process, which creates a foreign environment outside the body with extracorporeal circulation, shows its negative effects from the first minutes^[24]^. Cardiac arrest, especially after cardioplegia, causes serious global ischemia in the heart. The increased systemic inflammatory response in this process and the subsequent reperfusion period cause an increase in oxygen-derived free radicals, which can cause myocardial damage and many problems^[25]^. The primary targets of the formed ROS are the -SH groups of sulfur-containing amino acids (cysteine, methionine) in proteins. Reversible disulfide bonds form from -SH groups oxidized by ROS. This is the first indication of radical-mediated protein oxidation. Evidence has shown that there are significant relationships between changes in thiol/disulfide homeostasis and cardiovascular diseases^[4,25,26]^.

In some studies, significant positive correlations were found between the peak levels of troponin I — one of the most important cardiac biomarkers — and disulfide levels, disulfide/native thiol, and disulfide/total thiol ratios^[1]^. Altiparmak et al.^[27]^ reported that the native and total thiol levels were significantly decreased in patients with critical CAD, and these reductions were associated with the severity of CAD. These studies show that low native thiol levels are independent predictors of CAD.

In this study, albumin and IMA levels increased in plasma samples after CPB compared to pre-CPB, while native thiol, total thiol, and disulfide levels decreased. Accordingly, plasma native and total thiol levels decreased due to increased oxidative stress during CPB. *In vivo,* ROS played an active role in reducing the thiol level, and oxidative conversion and reduction of thiols to disulfides were performed. Under normal conditions, the disulfide level should increase in response to the decreasing native thiol level. However, the opposite was found in our study. The most important reason for this is that the level of disulfides in the CPB system may have decreased due to adherence to the non-endothelial surface and filters. During CPB, especially since capillary permeability is impaired, fluid flows from the vein to the tissues, and the intravascular fluid decreases. Accordingly, plasma albumin density may have increased after CPB.

Decreased myocardial blood flow causes hypoxia, acidosis, increase in reactive oxygen derivatives, and changes in serum albumin, thereby increasing the formation of IMA^[28]^. Similarly, in our study, plasma IMA levels increased after CPB. The reason for this may be the modification of albumin levels as a result of ischemia, acidosis, hypoxia, and ROS increase for many reasons, including impaired tissue perfusion, decreased blood flow, or foreign material surface during CPB, because even the slightest decrease in blood flow can change the level of IMA^[23]^. A study confirming this information has reported that continuous ventilation during CPB provides benefits for an increase in native and total thiol levels, a decrease in IMA levels, and a shorter hospital stay^[23]^. Continuous ventilation during CPB reportedly reduces ischemia and provides better inspiratory capacity by reducing lung damage^[29]^. IMA and redox homeostasis can be controlled by reducing ischemia and hypoxia with correct ventilation and oxygenation procedures during CPB.

Considered an ultrafiltrate of plasma, PF is also a transudate released from the cardiac interstitium, reflecting the cardiac interstitium’s composition and the production of macromolecules in the myocardium^[30,31]^. This study is the first in the literature to investigate IMA and thiol/disulfide homeostasis in PF, to support the view that PF is plasma ultrafiltrate, and to provide important information on the matter. While albumin, thiol, and disulfide were found to be lower in PF compared to plasma, IMA level was higher. It is important that thiol and IMA change inversely in the PF, which is closest to the heart tissue. The negative relationship between IMA and thiols in the PF indicates the effect of ischemia in the pericardium. Rapidly rising IMA levels are used as a cardiac biomarker in the early phase of ischemia and myocardial infarction^[17]^. Detection of IMA, which reflects ischemia in the myocardium and has a high importance in the PF, shows that the PF reflects the heart’s subclinical condition in CAD. However, it is difficult to measure the degree of subclinical conditions in the heart and their effect on the heart, and there is no clear procedure for treatment.

The relationship between albumin, IMA, and thiol/disulfide homeostasis parameters during and after CPB, postoperative complications, and the need for inotropic support have not been clarified. One study reported that thiol levels decreased in acute ischemic strokes and that thiol supplements could reduce neuronal damage associated with stroke and provide recovery. The same study reported that N-acetylcysteine or other thiol providers with antioxidant properties can also be used as a therapeutic intervention in the thiol/disulfide balance^[21,32]^.

The level of albumin, IMA, and thiol/disulfide homeostasis parameters during and after CPB are extremely important in terms of showing both the success of cardiovascular surgery and the effects of extracorporeal material-related complications. In this study, albumin and IMA levels increased, while native thiol, total thiol, and disulfide levels decreased in plasma samples after CPB compared with pre-CPB. The IMA-total thiol relationship in the PF supports the view that the PF is the plasma ultrafiltrate and provides important information about the course of the procedure. In the on-pump CABG, cardiac damage may be minimized by making therapeutic supplements with antioxidant properties to the thiol/disulfide balance or by developing biocompatible surfaces.

### Limitations

Our study has some limitations. This is a retrospective non-randomized study, and it included a limited number of patients. Since PF cannot be obtained from healthy people, free amino acid analysis could not be performed on them. Our main interest was to investigate the free amino acid profile in the PF and plasma of patients undergoing coronary angiography. Differently designed studies about the pathophysiological processes are needed to elucidate the underlying mechanisms of our findings.

## CONCLUSION

The extracorporeal circulation process in CPB is an unusual procedure for the body. The nonspecific inflammatory response shows its signs from the first minutes of CPB. In particular, the ischemia-reperfusion process during CPB has a negative effect. A possible relationship between dynamic thiol-disulfide homeostasis and CAD has been suggested. Disruption of the balance in thiol/disulfide homeostasis and increased IMA may provide information about the inflammatory response and damage due to the ischemia-reperfusion process in on-pump CABG. In this study, it is important to detect albumin, thiol, disulfide, and IMA in PF, similarly to plasma. This indicates that PF can be used for diagnostic purposes. The increase in IMA despite the decrease in thiol during CPB indicates the negative effect of extracorporeal circulation. Determination of IMA and thiol/disulfide homeostasis levels, which are predictive of postoperative complications in on-pump CABG, is important for the success of the surgical procedure and the prognosis of the disease.

## Abbreviations, Acronyms & Symbols

**ABSU** =: **Absorbance units**
**CABG** =: **Coronary artery bypass grafting**
**CAD** =: **Coronary artery disease**
**CPB** =: **Cardiopulmonary bypass**
**IMA** =: **Ischemia-modified albumin**
**PF** =: **Pericardial fluid**
**ROS** =: **Reactive oxygen species**
**SD** =: **Standard deviation**

## References

[1] Kundi H, Ates I, Kiziltunc E, Cetin M, Cicekcioglu H, Neselioglu S (2015). A novel oxidative stress marker in acute myocardial infarction;
thiol/disulphide homeostasis. Am J Emerg Med.

[2] Demirbag R, Rabus B, Sezen Y, Taskin A, Kalayci S, Balkanay M (2010). The plasma and tissue oxidative status in patients with coronary
artery disease: oxidative stress and coronary artery disease. Turkish J Thorac Cardiovasc Surg.

[3] Dzau VJ, Antman EM, Black HR, Hayes DL, Manson JE, Plutzky J (2006). The cardiovascular disease continuum validated: clinical evidence
of improved patient outcomes: part I: pathophysiology and clinical trial
evidence (risk factors through stable coronary artery
disease). Circulation.

[4] Topal FE, Karakaya Z, Akyol PY, Payza U, Çalişkan M, Topal F (2019). Increased thiol/disulphide ratio in patients with ST
elevation-acute coronary syndromes. Cukurova Med J.

[5] Dröge W (2002). Free radicals in the physiological control of cell
function. Physiol Rev.

[6] Erel O, Neselioglu S (2014). A novel and automated assay for thiol/disulphide
homeostasis. Clin Biochem.

[7] Otal Y, Demircan S, Sener A, Alisik M, Tanriverdi F, Haydar FGE (2018). Acute renal failure and thiol-disulfide
homeostasis. J Nephrol Ther.

[8] Ercan Haydar FG, Otal Y, Şener A, Pamukçu Günaydin G, Içme F (2020). The thiol-disulphide homeostasis in patients with acute
pancreatitis and its relation with other blood parameters. Ulus Travma Acil Cerrahi Derg.

[9] Erel Ö, Erdoğan S (2020). Thiol-disulfide homeostasis: an integrated approach with
biochemical and clinical aspects. Turk J Med Sci.

[10] Biswas S, Chida AS, Rahman I (2006). Redox modifications of protein-thiols: emerging roles in cell
signaling. Biochem Pharmacol.

[11] Circu ML, Aw TY (2010). Reactive oxygen species, cellular redox systems, and
apoptosis. Free Radic Biol Med.

[12] Sbarouni E, Georgiadou P, Voudris V (2011). Ischemia modified albumin changes - review and clinical
implications. Clin Chem Lab Med.

[13] Gaze DC (2009). Ischemia modified albumin: a novel biomarker for the detection of
cardiac ischemia. Drug Metab Pharmacokinet.

[14] Worster A, Devereaux PJ, Heels-Ansdell D, Guyatt GH, Opie J, Mookadam F (2005). Capability of ischemia-modified albumin to predict serious
cardiac outcomes in the short term among patients with potential acute
coronary syndrome. CMAJ.

[15] deFilippi C, Yoon S, Bounds C, Romar L, Ro A, Herzog W (2003). Early detection of myocardial ischemia by a novel blood based
biomarker: the kinetics of ischemia modified albumin. J Am Coll Cardiol.

[16] Roy D, Quiles J, Aldama G, Sinha M, Avanzas P, Arroyo-Espliguero R (2004). Ischemia modified albumin for the assessment of patients
presenting to the emergency department with acute chest pain but normal or
non-diagnostic 12-lead electrocardiograms and negative cardiac troponin
T. Int J Cardiol.

[17] Thielmann M, Pasa S, Holst T, Wendt D, Dohle DS, Demircioglu E (2017). Heart-type fatty acid binding protein and ischemia-modified
albumin for detection of myocardial infarction after coronary artery bypass
graft surgery. Ann Thorac Surg.

[18] Sinha MK, Roy D, Gaze DC, Collinson PO, Kaski JC (2004). Role of “Ischemia modified albumin”, a new biochemical marker of
myocardial ischaemia, in the early diagnosis of acute coronary
syndromes. Emerg Med J.

[19] Desborough JP (2000). The stress response to trauma and surgery. Br J Anaesth.

[20] Laffey JG, Boylan JF, Cheng DC (2002). The systemic inflammatory response to cardiac surgery:
implications for the anesthesiologist. Anesthesiology.

[21] Ellman G, Lysko H (1979). A precise method for the determination of whole blood and plasma
sulfhydryl groups. Anal Biochem.

[22] Bar-Or D, Lau E, Winkler JV (2000). A novel assay for cobalt-albumin binding and its potential as a
marker for myocardial ischemia-a preliminary report. J Emerg Med.

[23] Ozgunay SE, Ozsin KK, Ustundag Y, Karasu D, Ozyaprak B, Balci B (2019). The effect of continuous ventilation on thiol-disulphide
homeostasis and albumin-adjusted ischemia-modified albumin during
cardiopulmonary bypass. Braz J Cardiovasc Surg.

[24] Hatemi AC, Çeviker K, Tongut A, Özgöl I, Mert M, Kaya A (2016). Oxidant status following cardiac surgery with
phosphoryicholine-coated extracorporeal circulation systems. Oxid Med Cell Longev.

[25] Kundi H, Erel Ö, Balun A, Çiçekçioğlu H, Cetin M, Kiziltunç E (2015). Association of thiol/disulfide ratio with syntax score in
patients with NSTEMI. Scand Cardiovasc J.

[26] Bilir B, Akkoyun DC, Aydin M, Ozkaramanli Gur D, Degirmenci H, Albayrak N (2017). Association of coronary artery disease severity and
disulphide/native thiol ratio. Eur J Gen Med.

[27] Altiparmak IH, Erkuş ME, Sezen H, Demirbag R, Gunebakmaz O, Kaya Z (2016). The relation of serum thiol levels and thiol/disulphide
homeostasis with the severity of coronary artery disease. Kardiol Pol.

[28] Adly AAM, ElSherif NHK, Ismail EAR, Ibrahim YA, Niazi G, Elmetwally SH (2017). Ischemia-modified albumin as a marker of vascular dysfunction and
subclinical atherosclerosis in β-thalassemia major. Redox Rep.

[29] Chi D, Chen C, Shi Y, Wang W, Ma Y, Zhou R (2017). Ventilation during cardiopulmonary bypass for prevention of
respiratory insufficiency: a meta-analysis of randomized controlled
trials. Medicine (Baltimore).

[30] Fujita M, Komeda M, Hasegawa K, Kihara Y, Nohara R, Sasayama S (2001). Pericardial fluid as a new material for clinical heart
research. Int J Cardiol.

[31] Ben-Horin S, Shinfeld A, Kachel E, Chetrit A, Livneh A (2005). The composition of normal pericardial fluid and its implications
for diagnosing pericardial effusions. Am J Med.

[32] Karahan SC, Koramaz I, Altun G, Uçar U, Topbaş M, Menteşe A (2010). Ischemia-modified albumin reduction after coronary bypass surgery
is associated with the cardioprotective efficacy of cold-blood cardioplegia
enriched with N-acetylcysteine: a preliminary study. Eur Surg Res.

